# 

**Published:** 1876-08

**Authors:** 


INTERNATIONAL MEDICAL CONGRESS.—We have received, with request to publish, the following circular, which explains itself. From all we can learn, it will be one of the most important and interesting medical meetings ever held in this country:

The International Medical Congress will be formally opened at noon, on Monday, the fourth day of September.

The sessions of the Congress and of its Sections will be held in the University of Pennsylvania, Locust and Thirty-fourth streets.

The general meetings will be held daily, from 10 to 1 o'clock. The sections will meet at 1 o'clock.

Luncheon for members of the Congress will be served daily in the University building from 1 to 2 o'clock.

On Wednesday evening, September 6th, Dr. J. J. Woodward, U. S. A., will address the Congress on the scientific work of the Surgeon-General's Bureau.

The public dinner of the Congress will be given on Thursday evening, September 7th, at 7 o'clock.

The registration book will be open daily from Thursday, August 31st, to Saturday, September 2d, inclusive, from 12 to 3 P.M., in the hall of the College of Physicians, N. E. corner of Thirteenth and Locust Streets and at the University of Pennsylvania on Monday, September 4th, from 9 to 12 M., and daily thereafter from 9 to 10 A.M. Credentials must in every case be presented.

Letters addressed to the members of the Congress, to the care of the College of Physicians, N. E. corner Locust and Thirteenth Streets, Philadelphia, during the week of meeting, will be delivered at the University of Pennsylvania.

The Secretaries of State and Territorial Medical Societies are requested to forward without delay to the chairman of the committee on credentials, I. MINIS HAYS, M.D., 1607 Locust Street, Philadelphia, lists of their duly accredited delegates to the Congress.

Delegates and visitors intending to attend the Congress are earnestly requested individually to notify immediately the same committee.

This information is desired to facilitate registration, and to insure proper accommodation for the Congress.

Members intending to participate in the public (subscription) dinner of the Congress will please notify the Secretary of the Committee on Entertainment, J. EWING MEARS, M.D., 1429 Walnut Street, Philadelphia.

*Gentlemen intending to make communications upon scientific subjects, or to participate in any of the debates, will please notify the Commission before the fifteenth of August.*

PHILADELPHIA, July 20th, 1876.

---

THE AMERICAN DERMATOLOGICAL ASSOCIATION.—We have received the following letter, with the request that we call the attention of the profession to the contemplated meeting. We with pleasure insert the notice, as we feel that it is a move in the right direction, and one from which much good will result. The subject of skin diseases has received but little attention by the general practitioner of this country; the majority treating this class of affections without reference to classification or pathology, and, consequently, empirically.

If the contemplated meeting results in a permanent organization, as it certainly will, we hope in a short time to see its impress upon the medical colleges. While special diseases much less important have been made prominent in the curriculum of study in our medical schools, dermatology has in most of them been entirely ignored. We hope that the organization will be effected, and in a short time will so impress the profession with the importance of the subject that no school will dare present itself for patronage without giving a prominent place to skin diseases in its course of study:

NEW YORK, July . . . 1876.

DEAR SIR: At an informal meeting of the undersigned, held in Philadelphia, at the rooms of the Section of Practical Medicine of the American Medical Association, Wednesday, June

7th, 1876, after the election of a chairman and secretary pro tem, it was

*Resolved,* To call upon such American physicians as had evinced a special interest in dermatology to unite in forming an American Dermatological Association.

*Resolved,* That the meeting for organization be held in the University of Pennsylvania, Philadelphia, on Wednesday, September 6th, 1876, at 6 P.M., or immediately after the close of the meeting of the Section of Dermatology and Syphiology, of the International Medical Congress, on that day.

It is sincerely desired that you will be present and aid in the organization. Please signify your pleasure to the Secretary at the earliest opportunity, and oblige,

Very truly yours,

L. D. BULKLEY, *Secretary pro tem.,*
1 East 33d street, New York.
EDWARD WIGGLESWORTH, *Chairman,*
Boston, Mass.
LOUIS A. DUHRING, Philadelphia, Pa.
L. P. YANDELL, Jr., Louisville, Ky.
GEORGE HENRY FOX, New York.
J. E. ATKINSON, Baltimore, Md.

